# Composite Interlaminar Fracture Toughness Enhancement Using Electrospun PPO Fiber Veils Regulated by Functionalized CNTs

**DOI:** 10.3390/polym15153152

**Published:** 2023-07-25

**Authors:** Yuan Huang, Na Ning, Yiping Qiu, Yi Wei

**Affiliations:** 1 Key Laboratory of Textile Science & Technology, Ministry of Education, College of Textiles, Donghua University, 2999 North Renmin Road, Shanghai 201620, China; dorothy0522@foxmail.com (Y.H.); foreverluck7@sina.com (N.N.); 2 Center for Civil Aviation Composites, Donghua University, 2999 North Renmin Road, Shanghai 201620, China; 3 College of Textiles and Apparel, Quanzhou Normal University, Quanzhou 362046, China; ypqiu@dhu.edu.cn

**Keywords:** polyphenylene oxide, electrospun veil, CNTs, fracture toughness, epoxy composites

## Abstract

In this study, carbon nanotubes (CNTs) are functionalized through diazonium salt reaction to introduce polar groups onto their surfaces. These functionalized CNTs (FCNTs) are added into PPO solutions at different loadings (0 wt%, 0.5 wt%, 1 wt%, 1.5 wt%) and used for electrospinning. The results show that the addition of FCNTs facilitate the production of PPO veils having small fiber diameters. The veils are used as interleaves in CF/EP composite laminates. The Mode I and Mode II interlaminar fracture toughness tests reveal that PPO veils containing 0.5 wt% FCNTs exhibit the optimal toughening. *G_IC_^ini^* and *G_IIC_* have an improvement of approximately 120% and 180% over the untoughened samples, respectively, which is 15% and 26% higher than that of PPO veils containing no CNTs, respectively. The toughening mechanism is also analyzed using scanning electron microscopy (SEM).

## 1. Introduction

Composite materials, especially carbon fiber-reinforced epoxy (CF/EP) composites, are widely used due to their lightweight and high modulus [1,2]. However, their limited impact resistance is often considered a significant drawback of such materials [3]; hence, the toughening of composites has been a research hotspot. One important approach is resin matrix toughening which involves incorporating toughening agents, such as rubber or nanoparticles, into the resin matrix. These toughening agents act to absorb impact energy and to reduce cracks from propagating through the composite, thereby effectively enhancing their toughness. However, this approach often comes at the cost of reduced performance in terms of modulus and hot-wet *T_g_* [4]. Another approach is to insert toughener interlayers between adjacent fiber plies to enhance the interlaminar fracture toughness of CF/EP laminates [5,6], which has been proven to be one of the most effective toughening methods. The interlaminar toughening approach does not improve the fracture toughness of the matrix resin directly; rather, it acts as a mechanical reinforcement that deflects cracks, consuming more energy per unit area [7]. In addition, this interlayer of discrete phase can be made from a variety of forms including films [8], particles [9], and fibers [10]. Among these materials, electrospun fibers have gained approval as an interlaminar toughening agent for composites, owing to its high surface area to volume ratio and lightweight characteristics [11]. This technique improves the adhesion between the reinforcement fibers and the composite matrix without significantly increasing the final weight of the composite parts [12].

Dzenis and Renecker [13] were the pioneers who improved interlaminar fracture toughness, strength, and delamination resistance by incorporating nanofibrous veils into the interlayers, while maintaining the in-plane properties and weight. Subsequently, the utilization of polymer electrospinning techniques, including polycaprolactone (PCL) [14], polyvinyl alcohol (PVA) [15], polyacrylonitrile (PAN) [16], polysulfone (PSF) [17], and polyamide (PA) [18,19], in interlayer toughening of composite materials emerged as a popular approach. The research indicated that electrospun polymer interlayers can enhance the interlaminar fracture toughness (ILFT) of composite laminates via two distinct mechanisms. The first mechanism involves bridging the crack zones, wherein the nanofibers that remain intact within the resin rich layer containing the fibrous interleave hold together the newly formed surfaces as the crack propagates. The second mechanism is that the presence of the nanofibers causes the crack to travel along a longer and more complex path through the composite or resin, which increases the energy required for both crack initiation and propagation [20]. It is noteworthy that among the investigations of toughened composite materials with interlayered electrospun polymers, PA, which exhibits a highly superior toughening effect, has been the material of choice in most of the research. However, its tendency to absorb water restricts its applicability in the aerospace industry. Polyphenylene oxide (PPO) is a high-performance engineering thermoplastic with excellent thermal stability, mechanical strength, high *T_g_* and low water absorption. Previous research has shown that electrospun PPO could significantly improve toughness [21]. However, when using electrostatic spinning to prepare the fibers, PPO has limitations because of its low-polarity chemical structure, making it difficult to dissolve in polar solvents. In the process of electrospinning, uneven and coarse fibers caused by a charge imbalance are common problems [22]. The poor conductivity of PPO makes it a necessity to add conductive agents in the process of electrospinning to improve charge stability and the uniformity of fibers. However, research reports in this area are in short supply. Carbon nanotubes (CNTs) have excellent strength and electrical conductivity, and they have been extensively employed to enhance the electrical or mechanical properties of electrospun polymer fibers. Ra EJ et al. [23] found that the increase in CNT loading enhanced the conductivity of the polymer solution. Consequently, the generated larger currents during electrospinning resulted in the formation of smaller diameter fibers. Thus, the diameter of electrospun fibers strongly depends on the loading of CNTs. Moreover, there are numerous reports showing the enhancement of toughness via the incorporation of CNTs [24]. However, unmodified CNTs tend to aggregate in the electrospinning solution, which affects the uniformity and quality of the electrospun fibers.

This work aims to find an effective method for enhancing the electrospinning effect of PPO in order to create better toughening results and further validate the influence of the fiber diameter of the PPO veil on the toughening effect, which have not reported in the published materials. Based on the above literature review, the addition of CNTs to the PPO electrospinning solution may potentially enhance its conductivity. To address the dispersion issue of CNTs, we employed a diazonium salt reaction to introduce hydroxyl groups onto CNTs as a way of surface modification. To investigate the effect of CNT loading on PPO electrospinning, 0 wt%, 0.5 wt%, 1 wt%, and 1.5 wt% of the functionalized CNTs (FCNTs) were separately infused with PPO electrospinning solutions, corresponding to four veils: PPOv-0%, PPOv-0.5%, PPOv-1%, and PPOv-1.5%, with a fiber areal weight of 5 ± 0.5 g/m^2^. The effects of incorporating different loadings of FCNTs on PPO electrospinning were investigated. Subsequently, the veils of electrospun PPOv-0% and PPOv-FCNTs were embedded in a composite laminate, and their properties were evaluated according to Mode I and Mode II interlaminar fracture toughness. Finally, the mechanism of the toughening effect was investigated by observing and analyzing the fracture morphology under SEM.

## 2. Materials and Methods

### 2.1. Materials

Unidirectional carbon fiber prepreg UIN17500 (200 g/m^2^) and resin content of 35 ± 2 wt% was purchased from Weihai Guangwei Composites Co., Ltd. (Weihai, China). PPO 630 (Mn: 17,300, *T_g_*: 214 °C) was provided by SABIC Innovative Plastics. CNTs IM897P (diameter: 40–60 nm, length < 10 µm) were obtained from the Chengdu Institute of Organic Chemistry, Chinese Academy of Sciences. Chloroform, dimethyl formamide (DMF), aminophenyl ethanol, isoamyl nitrite, acetonitrile, and acetone were purchased from Sinopharm Chemical Reagent Co., Ltd. (Shanghai, China). The N_2_ was sourced from Air Liquide (Shanghai) Compressed Gas Co., Ltd. (Shanghai, China) and had purities exceeding 99.99%. All materials were used without further purification or treatment.

### 2.2. Preparation of Functionalized CNTs (FCNTs)

**Figure 1:**
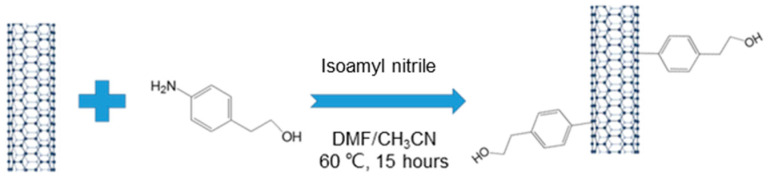
Scheme of surface functionalization of CNTs.

In a typical experiment, 0.1 g (8.32 mmol) of CNTs were immersed in 30 mL of N,N-dimethylformamide (DMF) and sonicated in an ultrasonic bath for two hours to obtain a CNT suspension. Separately, aminophenyl ethanol (4.56 g, 33.28 mmol) was dissolved in 30 mL of acetonitrile to prepare an arylamine solution in a closed container. Subsequently, the CNT suspension and arylamine solution were combined in a three-necked round-bottom flask and mixed under nitrogen purge for 30 min. Isoamyl nitrite (10.7 mL) was rapidly added via a dropping funnel. The resulting mixture was magnetically stirred and maintained at 60 °C for approximately 15 h under a nitrogen atmosphere in an air-tight environment. After cooling to room temperature, the suspension was filtered through a 0.45 μm polytetrafluoroethylene (PTFE) membrane and rinsed with DMF. The functionalized CNTs were further cleaned through repeated sonication and washing with DMF, followed by acetone. Finally, the functionalized CNTs were dried in a vacuum oven at 60 °C for 24 h. Figure 1 depicts the surface functionalization scheme of CNTs.

### 2.3. Preparation of the Electrospun Veils

**Figure 2:**
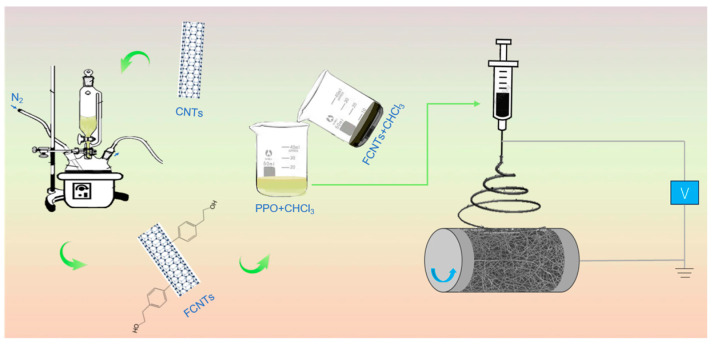
Schematic diagram of PPO electrospinning with the addition of FCNTs.

PPOv and PPOv-FCNTs electrospun veils were prepared using an electrospinning apparatus equipped with a standard nozzle. In the initial stage, FCNTs (0, 0.5, 1, and 1.5 wt%) were dispersed in chloroform, which is recognized as the optimal solvent for PPO, using a combination of magnetic stirring and sonication (SK7200B) set at a frequency of 35 kHz. Ultrasonic agitation was maintained for a duration of 30 min to achieve uniformity and stable dispersion. Subsequently, PPO particles were introduced into the above dispersion to obtain PPO/FCNTs mixtures with a PPO concentration of 18 wt% based on its electro spinnability. The mixtures were subjected to magnetic stirring for 12 h at room temperature to enable PPO to dissolve completely in chloroform. The electrospinning apparatus was located inside an environmental enclosure containing silica gel desiccant, which maintained a humidity level of 50% at an ambient temperature of approximately 26 °C. A potential difference of 18 kV was applied between the spinneret and the collector to electrospin the mixture. The solution was delivered through a 20 G (0.58/0.88 mm inner/outer diameter) stainless steel needle at a fixed feed rate of 0.5 mL/h to produce the veil. The distance between the spinneret and collector was set to 18 cm, and the collection was carried out using a rotating mandrel collector at a speed of 500 r/min to ensure uniform fiber deposition onto the aluminum foil. A schematic diagram of the process is shown in Figure 2. The collection time was adjusted to control the areal density of the veil. Based on previous research, a veil loading of 5 wt% (4.5 ± 0.5 g/m^2^) has been identified as the recommended concentration for effective interlaminar toughening; these veils have an area of about 250 × 220 mm^2^, and the areal density of the veil is controlled at 5 ± 0.5 g/m^2^.

### 2.4. Fabrication of Composite Panels and Test Coupons

Composite panels measuring 250 × 220 mm^2^ were fabricated for both the double cantilever beam (DCB) and end notch flexural (ENF) tests, using a prepreg lay-up of [0°]_20_. The stacks were cured by vacuum bag molding at a temperature of 120 °C for 90 min, as suggested by the prepreg manufacturer. The various types of veils were deposited on the mid-plane, where a PTFE film with 13 μm thickness was also located to act as the initial crack.

### 2.5. Characterizations

Mode I and Mode II fracture toughness tests were carried out on an Instron machine with a 2 kN load cell. The load-displacement measurements were taken at a displacement rate of 1 mm/min. A total of 5 specimens per sample were tested, and their average values were utilized for analysis.

According to ASTM D5528, white paint was applied to one side of the DCB test specimens, and 1 mm interval markings were made from the insertion point of the PTFE (Polytetrafluoroethylene) film up to a distance of *a*_0_ = 50 mm. Upon completion of the first increment of delamination growth, the specimens were unloaded. The Mode I crack initiation energy (*G_IC_^ini^*) was determined by applying a 5% offset to the maximum load, which was calculated using the intersection of the load-displacement curve, at the point when it became nonlinear, with a line drawn from the origin and offset by a 5% increase in compliance from the original linear region. In cases where the intersection occurred after the maximum load point, the maximum load was used to calculate this value. The specimens were then reloaded, without removal from the test machine jaws, until a final delamination length was attained. The second loading procedure was implemented to induce a natural Mode I pre-crack in the DCB specimen, which was defined as the Mode I pre-crack energy (*G_IC_^pre^*). The Mode I interlaminar fracture toughness *G_IC_* was subsequently determined by applying Equation (1) in accordance with modified beam theory:

(1) $$G_{IC}=\frac{3P_{c}\delta }{2b(a+\left|∆\right|)}$$

The test specimens had a width of *b* = 20 mm and an untoughened thickness of *h* = 3.1 mm. After adding PPO veils, the maximum increase in laminate thickness was approximately 9%. The total span length, *L*, was 150 mm, and the initial crack length was *a*_0_ = 50 mm. The applied load was denoted by *P_c_*, and the load point displacement was represented by *δ*. The crack length was denoted by *a*, and the crack length correction factor, ∆, could be experimentally determined by generating a least-square-plot of the cube root of compliance, *C*^1/3^, as a function of delamination length.

For the ENF test, the load-displacement curve was captured during the experiment, and a reduction in load was observed upon crack propagation. The initial maximum load and the corresponding displacement were recorded to determine the Mode Ⅱ interlaminar fracture toughness *G_IIC_* values using Equation (2). The data-reduction method used was in accordance with Russell and Street’s approach [25], as previously utilized by H. Albertsen [26] and Seyhan [27]. This involved the calculation of the critical strain energy release rate.

(2) $$G_{IIC}=\frac{9P_{c}\delta a^{2}}{2b(2L^{3}+3a^{3})}$$

where *L* is half the span length, 50 mm; *b* is the specimen width; and *a* is the crack length. The dimensions of test specimens were *b* = 20 mm; thickness of untoughened specimen *h* = 3.1 mm; and initial crack length *a*_0_ = 30 mm.

Fracture surface characterization was conducted using SEM performed on a Neo Scope JCM-6000Plus by Jeol Ltd. (Tokyo, Japan).

### 2.6. Data Analysis

The fiber diameter distribution was obtained by measuring the diameters of 120 randomly selected fibers in SEM photographs. The diameter distribution curve and average diameter were obtained by Gaussian fitting. All the data are presented as mean ± standard deviation (SD) of the mean.

## 3. Results and Discussion

### 3.1. Characterization of the FCNTs

**Figure 3:**
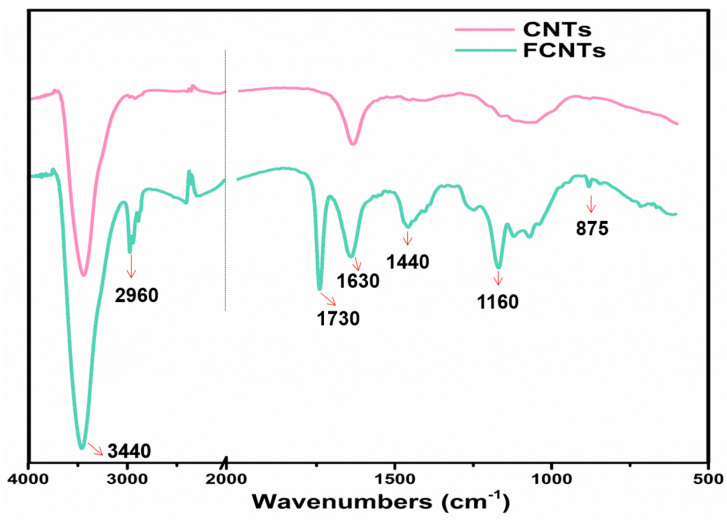
FTIR spectra of CNTs and FCNTs.

The FTIR spectra of the CNTs and the FCNTs are shown in Figure 3. It is shown that the FCNTs had a wider peak around 3446 cm^−1^, which was attributed to the stretching vibration of -OH, and the absorption peak at 1440 cm^−1^ was the bending vibration peak of -OH [28]. The absorption peak of 875 cm^−1^ corresponded to the para substitution peak of the benzene ring. The characteristic benzene ring stretching bands were observed at 2960 cm^−1^ (aromatic C–H stretching), 1630 cm^−1^ (aromatic ring stretching), and 1160 cm^−1^ (aromatic C–O stretching) [29], which confirmed the successful modification of CNTs.

**Figure 4:**
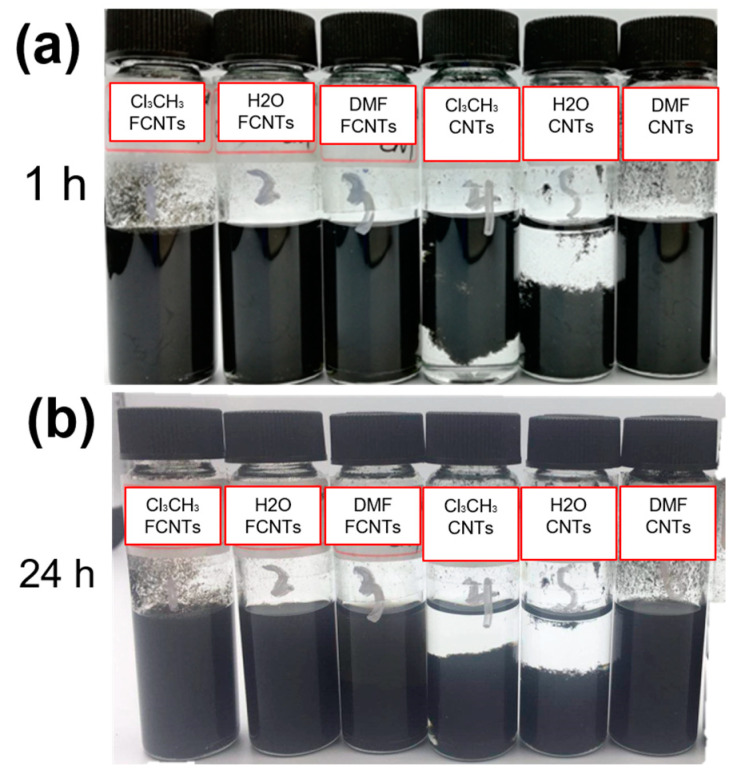
Digital photographs of FCNT (left three) and pristine CNTs (right three) dispersed in three solvents for (**a**) 0 h, (**b**) 24 h.

To examine the stability of the carbon nanotube dispersions, CNTs and FCNTs in three solvents: water, chloroform, and DMF, are illustrated in Figure 4. It is apparent that the CNTs undergo significant sedimentation within 1 h in water and chloroform, and more pronounced sedimentation is observed after 24 h. In contrast, the dispersions of FCNTs are relatively stable in all three solvents, and sedimentation is not observed in 24 h due to the chemical affinity between the polar modification groups and organic solvents, demonstrating the effective modification of the CNTs.

### 3.2. Morphology of PPO Veils Containing FCNTs

**Figure 5:**
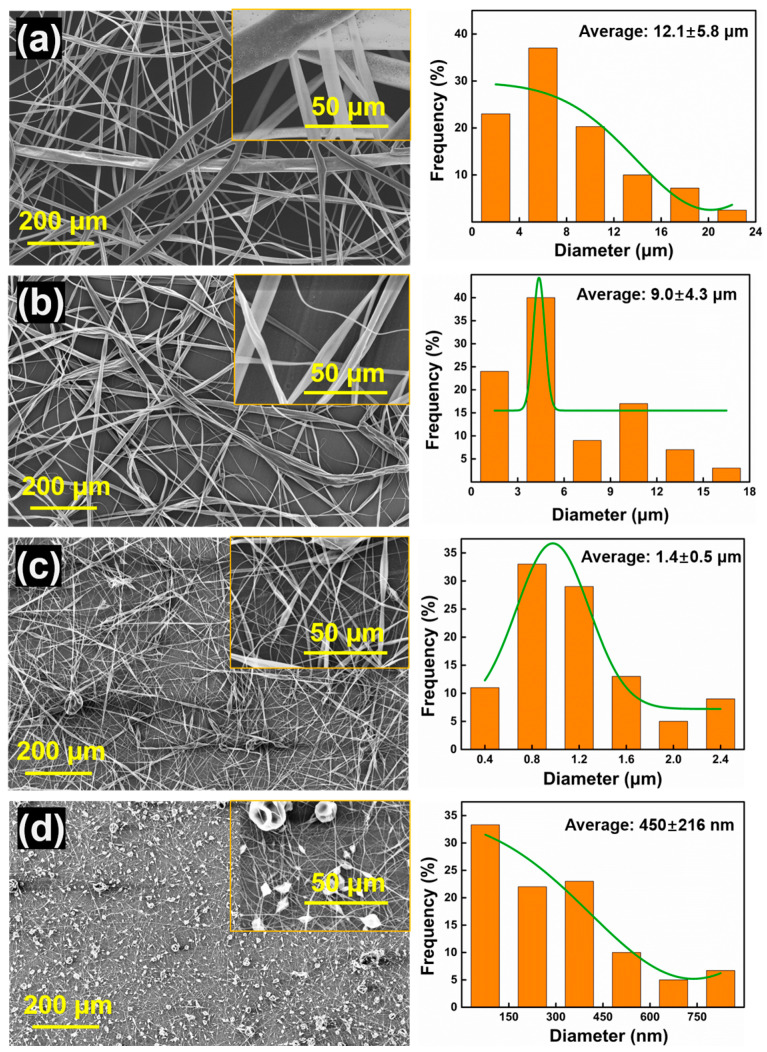
SEM micrograph and diameter distribution of PPOv-0% veil (**a**); PPOv-0.5% veil (**b**); PPOv-1% veil (**c**); PPOv-1.5% veil (**d**).

The SEM of PPOv-0%, PPOv-0.5%, PPOv-1%, and PPOv-1.5% veils are shown in Figure 5. It shows that the uniformity of PPO electrospun fibers is poor. At elevated concentrations of FCNTs, the resulting fibers exhibited reduced diameters and an increased number of knot-like structures within their morphology compared to those produced at lower concentrations. As the diameter of the fibers decreased, so did the dimensions of the knots form along their axis. The fiber diameter distribution graph indicates that the average diameter of the PPOv-0% veil is 12.1 ± 5.8 µm, while that of the PPOv-0.5% veil is 9.0 ± 4.3 µm, the PPOv-1% veil is 1.4 ± 0.5 µm, and the PPOv-1.5% veil is 450 ± 216 nm. The results indicate that with an increase in conductive materials in the electrospinning solution, it can reduce the diameter of PPO electrospun fibers effectively, which provides a good insight into the modification of PPO electrospinning. However, although the addition of FCNTs significantly improves the fineness of the fibers, as the loading of FCNTs increases, it causes a gradual increase in the relative number of knot structures among the fibers. This indicates that the incorporation of FCNTs has two distinct impacts on the formation of nanofibers. The first effect is an increase in the conductivity of the mixture, and the second effect is that it disrupts the homogeneity of the liquid. Consequently, the high viscosity and the agglomeration of FCNTs would lead to the formation of knots [30].

### 3.3. Mode I Interlaminar Fracture Toughness

**Figure 6:**
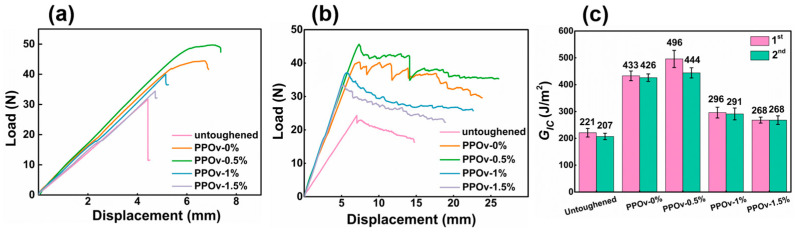
DCB test results: (**a**) load-displacement curves of first loading; (**b**) load-displacement curves of second loading; (**c**) values of *G_IC_^ini^* and *G_IC_^pre^*.

The DCB load-displacement curves of composites composed of the untoughened base material and along with the incorporated PPOv and PPOv-FCNTs veils under the 1st and 2nd loading conditions is shown in Figure 6a,b, respectively. The initial fracture toughness value (*G_IC_^ini^*) was deduced from the first loading curves. From Figure 6a,b, it can be observed that the fracture toughness improves with added PPOv and PPOv-FCNTs veils, while the PPOv-0.5% veil exhibits the highest toughness. From the first loading curve, it is also evident that the untoughened sample fractures with a sudden drop, indicating brittle fracture of the resin. On the other hand, the PPOv-0% and PPOv-0.5% veil-toughened samples exhibit a significant difference in the load-displacement behavior compared to other samples. Around the peak load, there are variations in displacement at the load point, with *P_c_* showing a slight upward trend. This indicates that the PPOv-0% and PPOv-0.5% veils effectively delay the onset of crack propagation while simultaneously controlling the rate of crack extension [31]. In the second loading cycle, it is observed that laminates modified with different veils exhibit nonlinear and irregular behaviors, resembling the untoughened system. However, as the crack propagates, the load on the untoughened sample decreases smoothly, whereas the veil-toughened samples demonstrate multiple crack initiations and crack arresting mechanisms, as well as moderate load drop and cyclic increases. This phenomenon is particularly pronounced in the PPOv-0% and PPOv-0.5% veil-toughened samples, which can likely be attributed to the bridging effect of the veil fibers.

In Figure 6c, it can be observed that the *G_IC_^ini^* and *G_IC_^prop^* of the PPOv-0.5% sample reaches 496 J and 444 J, respectively, which is approximately 120% and 110% higher than that of the untoughened sample, respectively, and nearly 15% and 4% higher than the sample toughened with PPOv-0% veil, respectively, which was noted to have a significant toughening effect in previous reports. This suggests that effective modification of PPO electrospinning can further enhance its toughening effect in composites. However, with an increase in FCNT loading, there is a decreasing trend in the *G_IC_* values. This trend may be attributed to the increased occurrence of knots in the veils resulting from the higher loading of FCNTs.

**Figure 7:**
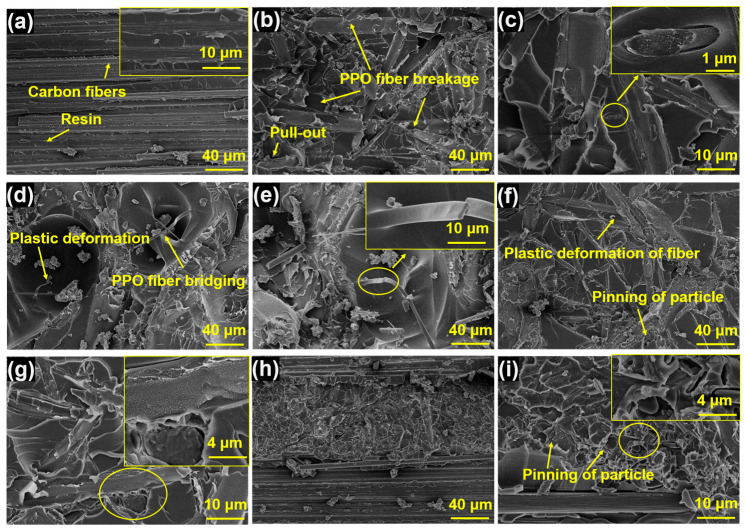
SEM images of fracture surfaces of DCB specimen at different magnifications: (**a**) untoughened laminates, laminates interleaved with (**b**,**c**) PPOv-0% veil; (**d**,**e**) PPOv-0.5% veil; (**f**,**g**) PPOv-1% veil; (**h**,**i**) PPOv-1.5% veil. The circles indicate the location of the local magnification.

Figure 7 illustrates the fracture surfaces of Mode I fracture toughness of five different samples. Figure 7a represents an untoughened sample characterized by a clean and smooth surface of the carbon fibers, indicating weak interfacial bonding. This suggests that cracks during the delamination process can propagate through the interfacial region between the carbon fiber and epoxy resin, resulting in adhesive failure. Consequently, the value of *G_IC_* is comparatively low. Figure 7b–e displays the surfaces of the PPOv-0% and PPOv-0.5% veil-toughened samples, respectively. Notably, the surfaces of these specimens exhibit prominent roughness, obscuring the visibility of carbon fibers and indicating stronger interaction [32]. Specifically, the surface analysis in Figure 7b reveals a significant incidence of PPO fiber breakage and pull-out. At a higher magnification, Figure 7c clearly shows the process of debonding and localized plastic deformation of PPO fibers, indicating the potential occurrence of fiber bridging during the loading process. The PPO veil network increases energy absorption by enlarging the plastic zone generated through the interleaving region, effectively impeding crack propagation [33]. Compared to the PPOv-0% veil surface, the interleaved surfaces in Figure 7d,e appear to be rougher, indicating a more tortuous path for crack propagation during the delaminating. During this process, the PPOv-0.5% fibers undergo bridging and plastic deformation, which corresponds to its outstanding fracture toughness.

Figure 7f–i respectively presents the surface views of toughened samples containing the PPOv-1% and PPOv-1.5% veils. The results shown in Figure 6 reveal that the toughening effect was less pronounced when the FCNT loading in the veil increased to 1 wt%. The surface roughness of the samples in Figure 7f,h exhibited a noticeable decrease compared to Figure 7b,d. Additionally, the fracture surfaces exhibited not only fibrous PPO but also particulate PPO formed by knots on the veil. With the same veil areal density, a higher number of knots implies a lower fiber content. Our previous studies have shown that, while the incorporation of PPO particles contributes to toughening, PPO in veil forms offers more substantial benefits. This observation provides a robust explanation for the results obtained regarding Mode I fracture toughness. The incorporation of 0.5% FCNTs into the PPO electrospinning mixture yields finer fibers, thereby resulting in an enhancement of fracture toughness. However, as the FCNT loading further increases, although a finer fiber diameter is achieved in the veil, the toughening effect relative to the pure PPOv veil is diminished. This is because finer fibers can provide better fracture toughness, but as the knot fraction in the veil increases, the fiber fraction decreases, thereby reducing the toughening effect.

### 3.4. Mode II Interlaminar Fracture Toughness

**Figure 8:**
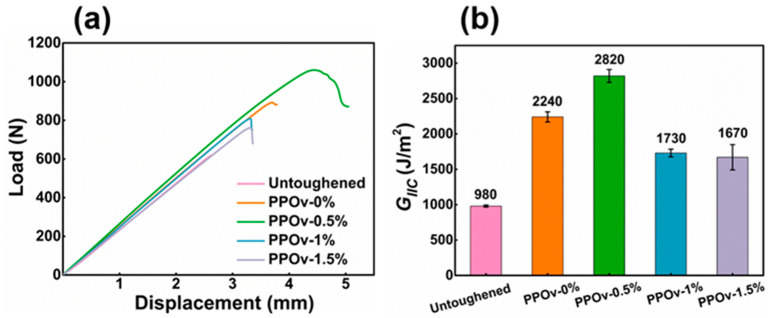
ENF test results: (**a**) load-displacement curves; (**b**) values of *G_IIC_*.

Typical load-displacement curves for untoughened composites and composites toughened with PPOv and PPOv-FCNTs veils are presented in Figure 8a. Similar to observations in Figure 6a, the load on the untoughened sample exhibits a steep decline (the testing machine was programmed to halt when the unloading exceeded 50 N for instrument safeguarding; as a result, the data recording abruptly ceased, and no downward trend was observed on the curve), indicating rapid crack propagation once the initial crack forms. The peak loads of samples containing veils are significantly higher than that of the untoughened base material, particularly in the case of the PPOv-0.5% veil-toughened sample. Moreover, the failure due to crack propagation differs significantly from that observed in the untoughened sample. The curves reveal that the load in the PPOv-0.5% veil-toughened sample only experiences a slight decrease, forming a region of plastic deformation, indicating significant resistance to crack propagation. A similar trend is observed in the second-ranked samples toughened with pure PPOv veils. However, this characteristic is less prominent in samples toughened with PPOv-1% and PPOv-1.5% veils, which have relatively lower toughening effectiveness.

Figure 8b provides a visual representation of the *G_IIC_* data for each sample. The PPOv-0.5% veil-toughened sample exhibits a significant advantage, with a value of 2820 J, surpassing the untoughened sample by approximately 180% (compared to 980 J). Additionally, it demonstrates an improvement of approximately 26% compared to the PPOv-0% veils. Such an improvement is rare compared to the already excellent base. Similar to the findings depicted in Figure 6c, the increase in FCNT loading, despite reducing the diameter of the veils, does not lead to further enhancement in *G_IIC_*. In fact, it is inferior to the toughening achieved by veils without the addition of FCNTs.

**Figure 9:**
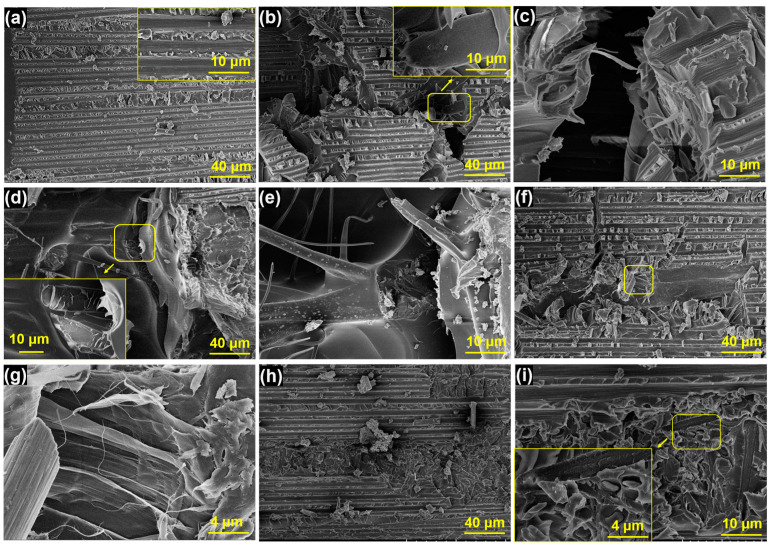
SEM images of fracture surface of ENF specimen at different magnifications: (**a**) untoughened laminates, laminates interleaved with (**b**,**c**) PPOv-0% veil; (**d**,**e**) PPOv-0.5% veil; (**f**,**g**) PPOv-1% veil; (**h**,**i**) PPOv-1.5% veil. The rectangles indicate the location of the local magnification.

Figure 9 displays the ENF fracture surfaces. Figure 9a reveals the relatively flat fracture morphology of the untoughened laminate, where cracks do not penetrate the resin-rich interlayers. The predominant failure mode involves fiber debonding and resin shear failure. In contrast, the veil-reinforced laminates exhibit a comparatively rougher appearance, as depicted in Figure 9b–i. Among them, Figure 9b,d,f,h represents low-magnification fracture surfaces, where noticeable fiber fractures caused by shear can be observed. Consistent with the ENF test results, higher values of *G_IIC_* correspond to more pronounced surface fractures, particularly evident in the cases of the PPOv-0% and PPOv-0.5% veils (Figure 9b,d), where the cracks penetrate through the interlayer of the laminate, indicating greater energy absorption during crack propagation. Furthermore, at higher magnifications (as shown in Figure 9b–e), both samples exhibit similar fracture surfaces characterized by a rough fracture matrix and pull-out, shear-damaged fibers. The fracture surfaces of samples toughened with PPOv-1% and PPOv-1.5% veils are shown in Figure 9f–i, respectively, where fiber pull-out, shear damage, and particle pinning can be observed.

## 4. Conclusions

FCNTs were demonstrated to be effective in increasing the conductivity of the PPO electrospinning mixture, therefore effectively reducing the diameter of the electrospun PPO fibers. However, its introduction at a high level also introduced unfavorable knots into the fibers; therefore, its level needs to be kept low. The Mode I and Mode II fracture toughness revealed that the PPOv-0.5 wt% FCNTs veil exhibited the highest toughening effect; *G_IC_^ini^* and *G_IIC_* reached 496 J and 2820 J, respectively, representing an improvement of approximately 120% and 180%, respectively, over the untoughened samples, and an additional enhancement of about 15% and 26%, respectively, over that of the PPOv veil. The toughening effect is remarkable and proves that the smaller fiber diameter of the PPO veil can provide a better toughening effect. However, a further increase in FCNT loading in the veil to above 1 wt% caused a reduction in the toughening effect due to the formation of PPO knots which consumed PPO that should have been converted into fibers. SEM analysis of the fracture surfaces revealed that the main toughening mechanisms involved fiber bridging, pull-out, plastic deformation, and shear damage.

## Data Availability

Data are contained within the article.
